# Ganglioneuroblastoma of the posterior mediastinum: a case report

**DOI:** 10.1186/1752-1947-5-322

**Published:** 2011-07-22

**Authors:** Saulat H Fatimi, Samira A Bawany, Awais Ashfaq

**Affiliations:** 1 Section of Cardiothoracic Surgery, Department of Surgery, Aga Khan University, Stadium Road, Karachi 74800, Pakistan; 2 Aga Khan University, Stadium Road, Karachi 74800, Pakistan

## Abstract

**Introduction:**

Ganglioneuroblastoma is a rare peripheral neuroblastic tumor that is derived from developing neuronal cells of the sympathetic nervous system, and is found mostly, but not exclusively, in babies and young children.

**Case presentation:**

To the best of our knowledge, there have been no previously reported cases of ganglioneuroblastoma of the mediastinum from Pakistan. We present a case of ganglioneuroblastoma in an eight-year-old Pakistani Sindhi boy incidentally found to have a large posterior mediastinal mass that on biopsy initially looked like ganglioneuroma. He underwent successful excision of the mediastinal mass and remained stable post-operatively. Final pathology findings showed a ganglioneuroblastoma. He has remained free of symptoms on long-term follow-up.

**Conclusions:**

The rarity of this tumor along with its almost exclusive occurrence in the pediatric population necessitates a thorough investigation of patients presenting with a symptomatic mass.

## Introduction

Ganglioneuroblastoma is a rare variety of peripheral neuroblastic tumor (neuroblastoma) that can arise anywhere along the sympathetic nervous system. It occurs almost exclusively in the pediatric population, with some reported cases in the adult population. It is the third most common childhood malignancy after leukemia and brain tumors, and is the commonest solid extracranial tumor among children [1]. Its true global incidence, however, is unknown. According to the Surveillance, Epidemiology and End Results (SEER) Registry maintained by the National Cancer Institute, the annual incidence of neuroblastoma is 7.6 per 1,000,000 population in the USA [2]. Of these cases, no statistics regarding the subtype of ganglioneuroblastoma are available. Here, we present the case of an eight-year-old previously healthy boy who presented to our clinic with non-specific signs and symptoms of pain and was later diagnosed to have ganglioneuroblastoma.

## Case presentation

**Figure 1:**
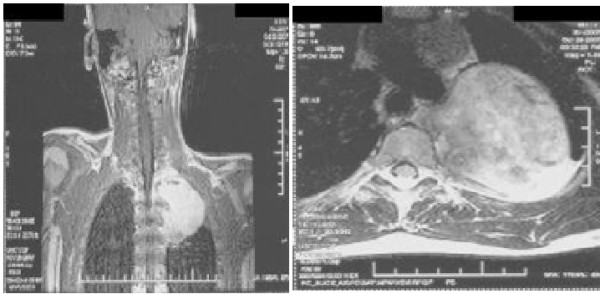
**MRI scan showing homogenous soft tissue density mass in the posterior mediastinum**. **(a) **Sagittal section. **(b) **Axial section.

An eight-year-old otherwise previously healthy Pakistani Sindhi boy presented to our clinic with complaints of right-sided lumbar pain for a week. At initial clinical assessment our patient's weight was 36.3 kg and his height was 142 cm. His overall nutritional status was good and had a normal height and build for his age with no history of weight loss. He had no other associated comorbidities. His family history was significant for diabetes mellitus, hypertension and untreated pancreatic cancer and renal cancer. The rest of his history and test results were unremarkable. A physical examination was grossly unremarkable. Ultrasound of the abdomen was performed, with normal results. A chest X-ray was performed that showed a homogenous soft tissue density mass in the posterior mediastinum with no evidence of rib erosion. To evaluate the mass further, an MRI was performed (Figure 1). The dimensions of the mass were 7.3 × 6.0 cm.

A computed tomography (CT)-guided biopsy of the mass was advised, which was carried out under general anesthesia. No complications were observed after the procedure and a post-procedural CT scan did not reveal any pneumothorax. Histopathology of the biopsy material showed multiple cones of tissue exhibiting spindle-shaped cells arranged haphazardly, having moderate cytoplasm and wavy spindle shaped nuclei. Scattered mature ganglion cells were also identified, characterized by abundant cytoplasm and round to oval nuclei; as such no immature element was identified nor was there any evidence of increased mitosis or areas of necrosis. The specimen was also treated with immunohistochemical stains and was found to be positive for S-100, synaptophysin, chromogranin, and leucocyte common antigen (LCA). These morphological and immunohistochemical findings were consistent with a diagnosis of ganglioneuroma, since no malignant features were identified.

In addition, whole body skeletal scintigraphy was performed with Tc-99 m for metastatic investigation, which showed bilateral symmetrical tracer distribution in both the axial and appendicular skeleton with no areas of abnormal tracer accumulation and normal excretion of the tracer from both kidneys and urinary bladder.

Bone marrow aspirate and trephine from the iliac crest performed for staging of the tumor showed all three cell lines with normal erythroid and myeloid precursors. The specimen was stained with hematoxylin and eosin and a few atypical mononuclear cells were noted; however, the sample was inadequate showing mainly cartilage, muscle and clot, and was hence non-diagnostic. There was nothing to suggest malignancy or granuloma in the sections examined. It was suggested to repeat an adequate length bone marrow trephine if there was a clinical indication to do so.

Surgery was planned for resection of tumor. Prior to that, pre-operative evaluation included a normal complete blood count, coagulation profile and serum lactate dehydrogenase levels. Surgery was approached using left thoracotomy. After entering the chest cavity, tumor was excised from the aorta, subclavian vessels and vertebral column. After adequate hemostasis, chest tubes were placed and routine closure of thoracotomy was carried out. The resected mass measuring 9.5 × 7 × 4.5 cm was sent for histopathology that confirmed the pre-operative diagnosis and presence of tumor-free margins.

Post-operatively, our patient remained hemodynamically stable. He received intravenous antibiotics and epidural marcaine infusion for pain relief, which was switched over to oral antibiotics and intravenous analgesics. Our patient was running a fever by the third post-operative day, which was followed by a transient episode of desaturation; however this improved without consequence. A post-operative chest X-ray was also unremarkable. On the fourth post-operative day our patient was discharged from the hospital on oral augmentin and non-steroidal anti-inflammatory drugs (NSAIDs) for pain. At discharge, the wound was clean, healing and healthy.

At follow-up a week later our patient's condition was stable. The final post-resection histopathology report showed a circumscribed and partially capsulated neoplasm composed of spindle cells arranged irregularly in short fascicles, scattered ganglion cells and abundant cytoplasm. Some nodules within the mass showed small, round neoplastic cells, scant cytoplasm, increased nuclear to cytoplasmic ration and hyperchromatic nuclei. The mitotic rate was identified as two to three cells per high-power field and the mitosis karyorrhexis index (MKI) was < 2%. Focal areas of hemorrhage and necrosis were also identified. Sections of tissues were also stained with immunohistochemical stains and were found to be positive for neurofilament, synaptophysin and chromogranin. The features showed that the tumor was a ganglioneuroblastoma, nodular type, which are composite Schwannian stroma-rich/stroma-dominant and stroma-poor element.

At a year later our patient had complaints of some bone pain for which a skeletal survey was performed and was found to be within normal limits. Our patient has thereafter remained stable.

## Discussion

Ganglioneuroblastoma is an uncommon peripheral neuroblastic tumor. The International Neuroblastoma Pathology Classification, popularly known as the Shimada system, grades these into four distinct categories based upon the histopathological balance between neural-type cells (primitive neuroblasts, maturing neuroblasts, and ganglion cells) and Schwann-type cells (Schwannian-blasts and mature Schwann cells). These include neuroblastomas (Schwannian stroma-poor), ganglioneuroblastoma intermixed (Schwannian stroma-rich), ganglioneuroma (Schwannian stroma-dominant) and ganglioneuroblastoma nodular (composite Schwannian stroma-rich/stroma-dominant and stroma-poor) and neuroblastic tumors unclassifiable [3].

Ganglioneuroblastoma have intermediate malignant potential, between that of neuroblastomas and ganglioneuromas. Histologically, they are considered malignant because they contain primitive neuroblasts along with mature ganglion cells. In contrast, their benign counterparts, ganglioneuromas are fully differentiated tumors that contain all mature cell types but lack the immature elements (such as neuroblasts), atypia, mitotic figures, intermediate cells, or necrosis [4].

Ganglioneuroblastomas occur with equal frequency in both the genders and most commonly in babies and in young children, with occurrence after 10 years of age being extremely rare [4]. They occur most commonly in the adrenal medulla, extra-adrenal retroperitoneum, and posterior mediastinum, with the neck and pelvis being less common sites of occurrence [5]. Thus, the occurrence of a posterior mediastianal ganglioneuroblastoma in our eight-year-old patient made this a rarity.

When dealing with pediatric masses, differentials such as rhabdomyosarcomas, Wilms tumor, germ cell tumors, and so on, should be taken into consideration. Rhabdomyosarcoma is the most common soft tissue sarcoma in children and usually originates in the head and neck (28%), extremities (24%), and genitourinary (GU) tract (18%). The cause is unclear; however, several genetic syndromes such as neurofibromatosis, Li-Fraumeni syndrome, and so on, are associated with it. Symptoms usually depend on the location and the tumours usually present as an expanding mass. Wilms tumor, by contrast, is the most common cancer of the kidneys in children. The majority are unilateral, encapsulated, vascularized, and usually do not cross the midline of the abdomen. They can go undetected early on because the tumor can grow large without causing pain. Children usually present with abdominal swelling or blood in the urine.

Patients with ganglioneuroblastoma often present clinically with pain caused by either the primary tumor or by metastatic disease. Patients with mediastinal tumors can present with stridor and shortness of breath secondary to tracheal deviation or narrowing. Large thoracic tumors can cause mechanical obstruction resulting in superior vena cava syndrome. Nerve or nerve root compression by the mass can result in peripheral neurological signs. Patients with cervical masses can present with Horner's syndrome [6]. However, in our patient these classical signs and symptoms were not present and the only complaint was that of non-specific lumbar pain.

Histological confirmation is required for definitive diagnosis. Tissue is obtained by incisional biopsy of the primary tumor or bone marrow trephine/aspirate in patients suspected of having metastatic disease in the bone marrow.

The most common site for metastasis in ganglioneuroblastomas is bone, which may mean patients present with limping and unexplained irritability (Hutchinson's syndrome). Another site of metastasis is the liver. Pepper syndrome is the presence of large liver metastases in babies such that intra-abdominal pressure becomes so high that there might be possibility of respiratory compromise. In children younger than a year old, skin metastases are common, which are darkly pigmented masses resembling blueberries (hence it is called 'blueberry muffin' syndrome) [7]. In our patient, investigation was performed to rule out metastasis and was found to be negative.

A CT scan is the imaging modality of choice to evaluate neuroblastic tumors. There is enough evidence to suggest that it is superior both in terms of determining tumor size and other characteristics including organ of origin, tissue invasion, vascular encasement, lymphadenopathy, and calcifications [5].

The prognosis for patients with localized disease and younger age is better; several screening programs were developed for the detection of neuroblastoma in infancy by measuring urinary catecholamines [8] but were not associated with any difference in mortality. As a result, they are not routinely recommended.

Age is taken into account when defining neuroblastoma histology as favorable or unfavorable. The younger the age at diagnosis, the better the survival rate [8] with children younger than a year old having considerably better survival than older children. Even the outcome of favorable or less aggressive neuroblastomas is worse in older children [9]. Hence, although it is not rare in the literature to find reports of good outcomes, the excellent results and successful follow-up in our patient is of significant note.

Mediastinal neuroblastomas are better in terms of prognosis than abdominal neuroblastomas in that patients with the former tend to present earlier when the size is still small, and hence complete resection of the tumor is possible [10]. Complete excision remains the mainstay of therapy of localized mediastinal neuroblastomas. There are no reports of the recommended duration of follow-up; however, follow-up is required with chest X-ray. The two-year event-free survival rates are 85% to 100%; relapses can be salvaged by further surgery or chemotherapy [11].

Differential markers for distinguishing ganglioneuromas from ganglioneuroblastomas at present are not available; immunohistochemical stains with antibodies such as neurofilament, synaptophysin, chromogranin, s-100 and LCA are generally positive in both ganglioneuromas and ganglioneuroblastomas [12]. However it has been shown on post-mortem histopathology specimens that an epidermal-growth-factor-like protein called delta-like (dlk), which regulates the differentiation of neuroblastoma cell lines, showed stronger expression in the ganglioneuroma cell lines and weaker expression in ganglioneuroblastoma cell lines [13], probably suggesting the loss of this differentiation factor in the progress toward the malignant disease.

## Conclusions

The rarity of ganglioneuroblastoma, along with its occurrence in the pediatric population, warrants a great deal of suspicion when a younger patient presents with a symptomatic mass. Given the wide variety of clinical symptoms the tumor can present with, it is essential that neuroblastoma remains as one of the differential diagnoses while working on such a case.

## Consent

Written informed consent was obtained from the patient's next-of-kin for publication of this case report and any accompanying images. A copy of the written consent is available for review by the Editor-in-Chief of this journal.

## Competing interests

The authors declare that they have no competing interests.

## Authors' contributions

SHF analyzed our patient's details and was primarily responsible for obtaining the full investigation results, including the surgery performed on our patient. SAB was involved in assisting the primary faculty, obtaining relevant details about the case and confirming its rarity, and was a major contributor to writing the manuscript. AA contributed a significant effort to writing the manuscript and editing the final draft. All authors read and approved the final manuscript.
